# Supplementary material for: A phosphoproteomic approach reveals that PKD3 controls PKA-mediated glucose and tyrosine metabolism
Source: Life Sci Alliance. 2021 Jun 18;4(8):e202000863. doi: 10.26508/lsa.202000863 (PMC8321662; doi:10.26508/lsa.202000863)
Supplement: Supplementary file 1 [file LSA-2020-00863_TableS1.docx]

### Supplementary Table 1

### Proteins identified by MS from IP with LxRxx[S*/T*] antibody

| **Uniprot entry** | **Protein names** | **Gene names** | **Peptides**  **EGFP** | **Peptides PKD3ca** | **norm.log2.Ratio.filt.LFQ.intensity** | **Sig.** | **Putative PKD substrate motifs** |
| --- | --- | --- | --- | --- | --- | --- | --- |
| Q5FWX6 | Serine/threonine-protein kinase D3 | Prkd3 | 0 | 46 | 9,56 | 2 | IlRvsS470 |
| Q9JKP5 | Muscleblind-like protein 1 | Mbnl1 | 1 | 2 | 7,73 | 2 |  |
| Q3UIL6 | Pleckstrin homology domain-containing family A member 7 | Plekha7 | 1 | 3 | 6,79 | 2 | IsRkyS229 VpRsiS562 LcResT766 LpReaT943 IiRhtS949 LeRlyS1060 |
| Q8QZS1 | 3-hydroxyisobutyryl-CoA hydrolase, mitochondrial | Hibch | 1 | 3 | 6,31 | 2 | LsRvsS15 |
| P97861 | Keratin, type II cuticular Hb6 | Krt86 | 2 | 12 | 6,29 | 2 |  |
| Q6A0A2 | La-related protein 4B | Larp4b | 1 | 13 | 5,68 | 2 | IlReiS246 VdRlpS628 |
| P62984 | Ubiquitin-60S ribosomal protein L40 | Uba52 | 9 | 6 | 4,94 | 2 |  |
| A2A7S8 | Uncharacterized protein KIAA1522 | Kiaa1522 | 3 | 20 | 4,37 | 2 | IqRrgS136 LtRpmS261 LgRfsS337 LpRppT477 LrRalS838 LeRpvS909 VaRkpS952 LpRteS967 |
| F8VQB6 | Unconventional myosin-X | Myo10 | 2 | 14 | 4,13 | 2 | IeRslS961 |
| Q8BHL4 | Retinoic acid-induced protein 3 | Gprc5a | 0 | 2 | 4,04 | 2 | LpRqrS275 |
| Q8K0Y2 | Keratin, type I cuticular Ha3-I | Krt33a | 2 | 6 | 3,93 | 2 |  |
| P11352 | Glutathione peroxidase 1 | Gpx1 | 8 | 10 | 3,50 | 2 |  |
| Q5SSZ5 | Tensin 3 | Tns3 | 1 | 12 | 3,50 | 2 | LiRwdS332 LlRkpS571 VqRgiS648 |
| Q05915 | GTP cyclohydrolase 1 | Gch1 | 2 | 7 | 3,45 | 2 |  |
| Q3UJB9 | Enhancer of mRNA-decapping protein 4 | Edc4 | 1 | 13 | 3,44 | 2 |  |
| P11370 | Retrovirus-related Env polyprotein from Fv-4 locus | Fv4 | 2 | 3 | 3,40 | 2 | LeRslT540 |
| P17918 | Proliferating cell nuclear antigen | Pcna | 0 | 2 | 3,27 | 2 | IcRdlS152 |
| Q640L5 | Coiled-coil domain-containing protein 18 | Ccdc18 | 1 | 2 | 3,26 | 2 | LeRnlS281 LdRllT695 LrRsiS1355 |
| P70275 | Semaphorin-3E | Sema3e | 4 | 11 | 3,21 | 2 |  |
| Q9JJY4 | Probable ATP-dependent RNA helicase DDX20 | Ddx20 | 3 | 6 | 3,19 | 2 | IiRnyT618 |
| P13020 | Gelsolin | Gsn | 9 | 14 | 3,07 | 2 |  |
| P28740 | Kinesin-like protein KIF2A | Kif2a | 1 | 5 | 3,03 | 2 |  |
| E9QN70 | Laminin subunit beta-1 | Lamb1 | 2 | 2 | 3,02 | 2 | LlRppS37 |
| F6ZDS4 | Nucleoprotein TPR | Tpr | 0 | 14 | 2,99 | 2 | LeRseT905 LqRasT1130 VeRpsT1881 |
| Q8BQ30 | Phostensin | Ppp1r18 | 5 | 15 | 2,91 | 2 | LeRrsS126 |
| O88487 | Cytoplasmic dynein 1 intermediate chain 2 | Dync1i2 | 3 | 4 | 2,86 | 2 | VeRalS212 |
| Q91YD3 | mRNA-decapping enzyme 1A | Dcp1a | 0 | 2 | 2,84 | 2 | LyrnaS121 LpRnsT359 LeRkaS542 |
| P35492 | Histidine ammonia-lyase | Hal | 21 | 25 | 2,81 | 2 | LvRshS195 |
| P28271 | Cytoplasmic aconitate hydratase | Aco1 | 1 | 4 | 2,68 | 2 | VmRfdT867 |
| Q9CXI3 | DBH-like monooxygenase protein 1 | Moxd1 | 0 | 4 | 2,54 | 2 | LtRcsS482 IyRpvT503 |
| Q8QZY1 | Eukaryotic translation initiation factor 3 subunit L | Eif3l | 2 | 4 | 2,50 | 2 | LlRlhS282  IqRtkS348 |
| P35564 | Calnexin | Canx | 2 | 4 | 2,49 | 2 |  |
| Q61699 | Heat shock protein 105 kDa | Hsph1 | 1 | 6 | 2,49 | 2 |  |
| Q8K2Q9 | Shootin-1 | Kiaa1598 | 4 | 24 | 2,46 | 2 |  |
| Q9D975 | Sulfiredoxin-1 | Srxn1 | 0 | 3 | 2,37 | 2 |  |
| Q91YU6 | Leucine zipper putative tumor suppressor 2 | Lzts2 | 1 | 4 | 2,36 | 2 |  |
| Q9QXL2 | Kinesin-like protein KIF21A | Kif21a | 5 | 26 | 2,36 | 2 | LqRlqT739 VlRrkT829 VtRklS855 LeRrvT933 IsRqsS1231 LkRfqS1482 |
| Q62266 | Cornifin-A | Sprr1a | 14 | 10 | 2,07 | 2 |  |
| Q6PEM6 | GRAM domain-containing protein 3 | Gramd3 | 4 | 2 | 1,98 | 2 | LsRdsT219 |
| P16331 | Phenylalanine-4-hydroxylase | Pah | 11 | 16 | 1,91 | 2 | LsRklS16  IpRpfS411 |
| Q9Z1Q9 | Valine--tRNA ligase | Vars | 4 | 9 | 2,29 | 1 |  |
| Q04750 | DNA topoisomerase 1 | Top1 | 5 | 4 | 2,25 | 1 |  |
| Q9CWY4 | Gem-associated protein 7 | Gemin7 | 0 | 2 | 2,21 | 1 |  |
| Q8K2D3 | Enhancer of mRNA-decapping protein 3 | Edc3 | 0 | 2 | 2,09 | 1 | VyRriT262 |
| P46062 | Signal-induced proliferation-associated protein 1 | Sipa1 | 0 | 6 | 2,04 | 1 | LlRsgS53 LpRtlS903 |
| Q91WK0 | Leucine-rich repeat flightless-interacting protein 2 | Lrrfip2 | 22 | 18 | 1,99 | 1 | LlRstS151 |
| Q91WT9 | Cystathionine beta-synthase | Cbs | 2 | 2 | 1,99 | 1 | IvRtpT190 |
| P61967 | AP-1 complex subunit sigma-1A | Ap1s1 | 1 | 2 | 1,98 | 1 |  |
| O35682 | Myeloid-associated differentiation marker | Myadm | 2 | 2 | 1,96 | 1 |  |
| Q3THS6 | S-adenosylmethionine synthase isoform type-2 | Mat2a | 4 | 5 | 1,96 | 1 | LrRngT172 |
| Q8CGK3 | Lon protease homolog, mitochondrial | Lonp1 | 5 | 4 | 1,93 | 1 |  |
| P09055 | Integrin beta-1 | Itgb1 | 2 | 3 | 1,92 | 1 |  |
| Q91VY9 | Zinc finger protein 622 | Znf622 | 2 | 2 | 1,91 | 1 | LpRavT410 VqRmkS452 |
| Q6IMF0 | Keratin, type II cuticular Hb3 | Krt83 | 2 | 13 | 1,81 | 1 |  |
| Q60953 | Protein PML | Pml | 3 | 7 | 1,76 | 1 | LdRnhS227 LqRirT332 LaRnmS748 |
| A2AN08 | E3 ubiquitin-protein ligase UBR4 | Ubr4 | 8 | 15 | 1,70 | 1 | LnRldS950 LtRmtT1472 IvRenS1503 VkRtpS1732 LvRhaS1760 LtRlaS1945 IeRapS2364 VmRllS3057 LaRhnT4938 |
| Q8VDJ3 | Vigilin | Hdlbp | 0 | 5 | 1,70 | 1 | VaRlqT149 ViRgpS706 |
| P84084 | ADP-ribosylation factor 5 | Arf5 | 3 | 4 | 1,68 | 1 |  |
| E9Q450 | Tropomyosin alpha-1 chain | Tpm1 | 64 | 56 | 1,67 | 1 |  |
| P20918 | Plasminogen | Plg | 3 | 14 | 1,64 | 1 | IpRctT264 LsRpaT678 |
| P42125 | Enoyl-CoA delta isomerase 1, mitochondrial | Eci1 | 4 | 4 | 1,61 | 1 |  |
| Q91XV3 | Brain acid soluble protein 1 | Basp1 | 1 | 3 | 1,59 | 1 |  |
| Q921U8 | Smoothelin | Smtn | 10 | 23 | 1,58 | 1 | VtRlgS521 VqRstS798 |
| Q9CQA6 | Coiled-coil-helix-coiled-coil-helix domain-containing protein 1 | Chchd1 | 1 | 2 | 1,58 | 1 |  |
| Q99NB9 | Splicing factor 3B subunit 1 | Sf3b1 | 2 | 2 | 1,57 | 1 |  |
| Q9DBJ3 | Brain-specific angiogenesis inhibitor 1-associated protein 2-like protein 1 | Baiap2l1 | 4 | 7 | 1,54 | 1 | LqRsvS332 |
| Q8CC35 | Synaptopodin | Synpo | 0 | 4 | 1,54 | 1 | LgRstS134 LaRcpS740 |
| Q91YW3 | DnaJ homolog subfamily C member 3 | Dnajc3 | 3 | 2 | 1,52 | 1 |  |
| Q0VG62 | Uncharacterized protein C8orf59 homolog | 1810022K09Rik | 2 | 2 | 1,52 | 1 | VpRpeT34 |
| P10648 | N-terminally processed;Glutathione S-transferase A2 | Gsta2 | 3 | 6 | 1,51 | 1 |  |
| Q62523 | Zyxin | Zyx | 0 | 2 | 1,50 | 1 |  |
| A0A087WQ89 | MCG5930 | 2210011C24Rik | 4 | 6 | 1,49 | 1 | LsRpgS48 |
| Q9D0R2 | Threonine--tRNA ligase, cytoplasmic | Tars | 0 | 3 | 1,44 | 1 |  |
| Q8K2I2 | Coiled-coil alpha-helical rod protein 1 | Cchcr1 | 0 | 4 | 1,44 | 1 | VeRmsT401 VaRipS459 |
| P35700 | Peroxiredoxin-1 | Prdx1 | 7 | 10 | 1,41 | 1 | IlRqiT143 |
| P07901 | Heat shock protein HSP 90-alpha | Hsp90aa1 | 10 | 17 | 1,39 | 1 | LlRyyT468 |
| Q8VE19 | WD repeat-containing protein mio | Mios | 6 | 12 | 1,38 | 1 |  |
| Q9CQ22 | Ragulator complex protein LAMTOR1 | Lamtor1 | 4 | 4 | 1,36 | 1 |  |
| Q91YD6 | Villin-like protein | Vill | 8 | 12 | 1,25 | 1 |  |
| P99029 | Peroxiredoxin-5, mitochondrial | Prdx5 | 6 | 5 | 1,23 | 1 | VlRasT21 |
| O70325 | Phospholipid hydroperoxide glutathione peroxidase, mitochondrial | Gpx4 | 4 | 5 | 1,23 | 1 |  |
| Q91ZU6 | Dystonin | Dst | 18 | 40 | 1,21 | 1 | LhRleS682 VaRkkS739 IqRkyS833 VeRwqS1272 LeRqdT1703 VlRpeS2146 LtRqkS3894 LtRskS4092 LlRslS4680 LdRakT5202 LtRqlS5407 LlRkqS5488 LeRaqS5759 VeRgrS6520 VpRagS7365 |
| Q9D0I9 | Arginine--tRNA ligase, cytoplasmic | Rars | 5 | 7 | 1,18 | 1 | IeRgeS336 |
| Q91YR1 | Twinfilin-1 | Twf1 | 17 | 13 | 1,16 | 1 | LfRldS75 |
