# Supplementary material for: A phosphoproteomic approach reveals that PKD3 controls PKA-mediated glucose and tyrosine metabolism
Source: Life Sci Alliance. 2021 Jun 18;4(8):e202000863. doi: 10.26508/lsa.202000863 (PMC8321662; doi:10.26508/lsa.202000863)
Supplement: Supplementary file 2 [file LSA-2020-00863_TableS2.docx]

**Supplementary Table 2**

**Proteins identified by MS from IP with Rxx[S*/T*] antibody**

| **Uniprot entry** | **Protein names** | **Gene names** | **Peptides**  **EGFP** | **Peptides PKD3ca** | **norm.log2.Ratio.filt.LFQ.intensity** | **Sig.** | **Putative PKD substrate motifs** |
| --- | --- | --- | --- | --- | --- | --- | --- |
| Q8BQZ4 | Ral GTPase-activating protein subunit beta | Ralgapb | 1 | 3 | 10,24 | 2 | LaResS156 IsRprS357 InRdnS470 LvRgmS971 |
| P63260 | Actin, cytoplasmic 2 | Actg1 | 48 | 44 | 8,04 | 2 |  |
| Q9JKP5 | Muscleblind-like protein 1 | Mbnl1 | 1 | 2 | 7,53 | 2 |  |
| P35700 | Peroxiredoxin-1 | Prdx1 | 3 | 11 | 7,19 | 2 | IlRqiT143 |
| K9J7B2 | UDP-glucuronosyltransferase | Ugt1a6b | 1 | 13 | 6,35 | 2 | VpRfyT197 LkRdvS242 ImRlsS443 |
| Q62426 | Cystatin-B | Cstb | 1 | 6 | 6,22 | 2 |  |
| P70275 | Semaphorin-3E | Sema3e | 1 | 14 | 5,85 | 2 |  |
| Q62266 | Cornifin-A | Sprr1a | 2 | 9 | 5,45 | 2 |  |
| Q5FWX6 | Serine/threonine-protein kinase D3 | Prkd3 | 3 | 42 | 5,38 | 2 | IlRvsS470 |
| P54823 | Probable ATP-dependent RNA helicase DDX6 | Ddx6 | 2 | 14 | 5,33 | 2 |  |
| P63168 | Dynein light chain 1, cytoplasmic | Dynll1 | 0 | 3 | 5,26 | 2 |  |
| P01887 | Beta-2-microglobulin | B2m | 1 | 2 | 5,24 | 2 |  |
| Q80TV8 | CLIP-associating protein 1 | Clasp1 | 0 | 30 | 5,04 | 2 | LeRhiS485 LnRplS568 LqRsrS600 LgRirT642 VsRiiT1026 IgRtpS1098 LrRsyS1159 ItRedS1333 IkRaqT1520 |
| O54962 | Barrier-to-autointegration factor | Banf1 | 1 | 3 | 5,00 | 2 |  |
| Q80U30 | Protein CLEC16A | Clec16a | 1 | 2 | 4,96 | 2 | IlRqkS89 |
| P12787 | Cytochrome c oxidase subunit 5A, mitochondrial | Cox5a | 0 | 3 | 4,95 | 2 |  |
| Q8VCH8 | UBX domain-containing protein 4 | Ubxn4 | 0 | 7 | 4,95 | 2 | IeRrkT230 VkResT310 IwRliS428 IyRlrT487 |
| P10810 | Monocyte differentiation antigen CD14 | Cd14 | 1 | 10 | 4,87 | 2 | LkRvdT71 LdRnpS308 |
| O09106 | Histone deacetylase 1 | Hdac1 | 0 | 8 | 4,83 | 2 | VaRcwT313 |
| Q9CXF4 | TBC1 domain family member 15 | Tbc1d15 | 0 | 3 | 4,81 | 2 |  |
| Q9CQQ7 | ATP synthase F(0) complex subunit B1, mitochondrial | Atp5f1 | 2 | 7 | 4,80 | 2 |  |
| P27773 | Protein disulfide-isomerase A3 | Pdia3 | 1 | 11 | 4,58 | 2 | LqReaT485 |
| P62821 | Ras-related protein Rab-1A | Rab1A | 1 | 4 | 4,53 | 2 | IdRyaS114 |
| P09528 | Ferritin heavy chain | Fth1 | 1 | 8 | 4,49 | 2 |  |
| Q9DBG1 | Sterol 26-hydroxylase, mitochondrial | Cyp27a1 | 1 | 11 | 4,47 | 2 | VsRdpS439 |
| Q8K0D0 | Cyclin-dependent kinase 17 | Cdk17 | 0 | 4 | 4,47 | 2 | LrRphS75 IhRriS137 LaRakS339 |
| Q8BHL4 | Retinoic acid-induced protein 3 | Gprc5a | 0 | 3 | 4,46 | 2 | LpRqrS275 |
| P20918 | Plasminogen | Plg | 2 | 12 | 4,39 | 2 | IpRctT264 LsRpaT678 |
| Q9R049 | E3 ubiquitin-protein ligase AMFR | Amfr | 0 | 12 | 4,34 | 2 | LqRqrT642 |
| Q05421 | Cytochrome P450 2E1 | Cyp2e1 | 1 | 15 | 4,27 | 2 |  |
| Q3UJB9 | Enhancer of mRNA-decapping protein 4 | Edc4 | 0 | 12 | 4,22 | 2 |  |
| Q91WT8 | RNA-binding protein 47 | Rbm47 | 2 | 7 | 4,13 | 2 | VqRipT533 |
| P11352 | Glutathione peroxidase 1 | Gpx1 | 4 | 12 | 4,13 | 2 |  |
| Q8BQ30 | Phostensin | Ppp1r18 | 4 | 23 | 4,09 | 2 | LeRrsS126 |
| Q9DCX2 | ATP synthase subunit d, mitochondrial | Atp5h | 1 | 3 | 4,06 | 2 |  |
| Q64282 | Interferon-induced protein with tetratricopeptide repeats 1 | Ifit1 | 1 | 13 | 4,06 | 2 |  |
| Q8R084 | UDP-glucuronosyltransferase | Ugt2b1 | 2 | 8 | 4,05 | 2 | LgRptT246 |
| P03975 | IgE-binding protein | Iap | 1 | 9 | 4,02 | 2 | VsRkrS107 |
| Q8VCW8 | Acyl-CoA synthetase family member 2, mitochondrial | Acsf2 | 1 | 7 | 3,93 | 2 |  |
| V9GX76 | Unconventional myosin-VI | Myo6 | 73 | 93 | 3,93 | 2 | VlRylT169 LnRgcT279 |
| Q8CHQ9 | Probable N-acetyltransferase CML2 | Cml2 | 2 | 7 | 3,92 | 2 |  |
| P61222 | ATP-binding cassette sub-family E member 1 | Abce1 | 1 | 5 | 3,91 | 2 |  |
| Q3TJZ6 | Protein FAM98A | Fam98a | 0 | 6 | 3,90 | 2 | IlRtsS283 |
| P14211 | Calreticulin | Calr | 1 | 10 | 3,88 | 2 |  |
| Q6PDK2 | Histone-lysine N-methyltransferase 2D | Kmt2d | 2 | 25 | 3,82 | 2 | LgRagT424 VlRnlT1560 LdRipT1837 lsRgqT2695 VsRppS2962 |
| Q9CY27 | Very-long-chain enoyl-CoA reductase | Tecr | 1 | 7 | 3,75 | 2 |  |
| Q02013 | Aquaporin-1 | Aqp1 | 1 | 3 | 3,75 | 2 | LeRnqT44 LtnfS207 |
| P62071 | Ras-related protein R-Ras2 | Rras2 | 1 | 7 | 3,73 | 2 |  |
| F8VQB6 | Unconventional myosin-X | Myo10 | 0 | 24 | 3,72 | 2 | IeRslS961 |
| P58660 | Caspase recruitment domain-containing protein 10 | Card10 | 4 | 9 | 3,68 | 2 | VrRvlS629 VeRgsS1011 |
| O88342 | WD repeat-containing protein 1 | Wdr1 | 1 | 3 | 3,68 | 2 | VeRgvS20 |
| Q91YN9 | BAG family molecular chaperone regulator 2 | Bag2 | 2 | 5 | 3,66 | 2 |  |
| Q99104 | Unconventional myosin-Va | Myo5a | 3 | 17 | 3,65 | 2 | IrRaaT841 |
| Q91YU6 | Leucine zipper putative tumor suppressor 2 | Lzts2 | 0 | 8 | 3,64 | 2 |  |
| P63242 | Eukaryotic translation initiation factor 5A-1 | Eif5a | 1 | 3 | 3,64 | 2 |  |
| Q9CQW9 | Interferon-induced transmembrane protein 3 | Ifitm3 | 1 | 3 | 3,64 | 2 |  |
| Q922F4 | Tubulin beta-6 chain | Tubb6 | 6 | 21 | 3,63 | 2 |  |
| Q8VCX1 | 3-oxo-5-beta-steroid 4-dehydrogenase | Akr1d1 | 1 | 2 | 3,61 | 2 |  |
| Q99KK2 | N-acylneuraminate cytidylyltransferase | Cmas | 0 | 12 | 3,61 | 2 | LaRggS53 VhRrsS111 |
| Q8BG80 | F-box only protein 46 | Fbxo46 | 0 | 8 | 3,61 | 2 | LyRhvS453 |
| Q61937 | Nucleophosmin | Npm1 | 2 | 5 | 3,60 | 2 |  |
| Q9JLJ2 | 4-trimethylaminobutyraldehyde dehydrogenase | Aldh9a1 | 3 | 10 | 3,58 | 2 |  |
| P56593 | Cytochrome P450 2A12 | Cyp2a12 | 0 | 12 | 3,55 | 2 | IpRriT373 |
| P58137 | Acyl-coenzyme A thioesterase 8 | Acot8 | 1 | 4 | 3,52 | 2 | VeRirT104 |
| Q9JHU4 | Cytoplasmic dynein 1 heavy chain 1 | Dync1h1 | 4 | 20 | 3,52 | 2 | IsRdlS387 IdRveT560 IdRqlT657 LpRiqS999 LeRerS1622 LaRlrS2382 IrRitT2521 VhRkyT2966 LeRmnT3008 LeRlfT4272 LpRswS4462 |
| Q63886 | UDP-glucuronosyltransferase 1-1 | Ugt1a1 | 2 | 12 | 3,51 | 2 | VkRdsS123 ImRlsS447 |
| Q99K48 | Non-POU domain-containing octamer-binding protein | Nono | 3 | 7 | 3,50 | 2 |  |
| Q8BHD7 | Polypyrimidine tract-binding protein 3 | Ptbp3 | 2 | 11 | 3,48 | 2 |  |
| Q922P9 | Putative oxidoreductase GLYR1 | Glyr1 | 1 | 6 | 3,46 | 2 |  |
| A2AIX1 | Protein transport protein sec16 | Sec16a | 0 | 17 | 3,44 | 2 | LtRapS2101 LsRcsS2315 |
| Q8BWS5 | G protein-regulated inducer of neurite outgrowth 3 | Gprin3 | 0 | 7 | 3,43 | 2 |  |
| Q91VY9 | Zinc finger protein 622 | Znf622 | 0 | 8 | 3,43 | 2 | LpRavT410 VqRmkS452 |
| Q3UMC0 | Spermatogenesis-associated protein 5 | Spata5 | 1 | 4 | 3,41 | 2 | VeRgsS739 |
| Q61699 | Heat shock protein 105 kDa | Hsph1 | 0 | 7 | 3,35 | 2 |  |
| Q61495 | Desmoglein-1-alpha | Dsg1a | 2 | 2 | 3,31 | 2 | VdRevT120 VfRpgS389 LqRtcT476 |
| Q9D2G2 | Dihydrolipoyllysine-residue succinyltransferase component of 2-OGDC, mitochondrial | Dlst | 1 | 8 | 3,28 | 2 | VsRafS13 |
| Q5U465 | Coiled-coil domain-containing protein 125 | Ccdc125 | 0 | 8 | 3,28 | 2 | VpRssS9 LkRscS492 |
| P17182 | Alpha-enolase | Eno1 | 1 | 9 | 3,28 | 2 |  |
| Q9CQF0 | 39S ribosomal protein L11, mitochondrial | Mrpl11 | 1 | 7 | 3,27 | 2 |  |
| P13020 | Gelsolin | Gsn | 6 | 10 | 3,27 | 2 |  |
| P26039 | Talin-1 | Tln1 | 1 | 10 | 3,26 | 2 | LnRcvS1201 |
| Q921G7 | Electron transfer flavoprotein-ubiquinone oxidoreductase, mitochondrial | Etfdh | 1 | 4 | 3,25 | 2 | VnRnlS550 |
| Q62186 | Translocon-associated protein subunit delta | Ssr4 | 1 | 4 | 3,25 | 2 |  |
| O35632 | Hyaluronidase-2 | Hyal2 | 1 | 3 | 3,20 | 2 | VyRqsS155 VrRnpS382 |
| P09803 | Cadherin-1 | Cdh1 | 1 | 4 | 3,19 | 2 |  |
| P08226 | Apolipoprotein E | Apoe | 1 | 12 | 3,17 | 2 |  |
| Q91ZU6 | Dystonin | Dst | 9 | 65 | 3,16 | 2 | LhRleS682 VaRkkS739 IqRkyS833 VeRwqS1272 LeRqdT1703 VlRpeS2146 LtRqkS3894 LtRskS4092 LlRslS4680 LdRakT5202 LtRqlS5407 LlRkqS5488 LeRaqS5759 VeRgrS6520 VpRagS7365 |
| Q6PGG6 | Guanine nucleotide-binding protein-like 3-like protein | Gnl3l | 0 | 2 | 3,16 | 2 |  |
| P35564 | Calnexin | Canx | 1 | 7 | 3,16 | 2 |  |
| O35295 | Transcriptional activator protein Pur-beta | Purb | 1 | 8 | 3,14 | 2 |  |
| P34914 | Bifunctional epoxide hydrolase 2 | Ephx2 | 1 | 4 | 3,14 | 2 |  |
| Q8BGH2 | Sorting and assembly machinery component 50 homolog | Samm50 | 0 | 8 | 3,14 | 2 | LsRtaS243 |
| Q64433 | 10 kDa heat shock protein, mitochondrial | Hspe1 | 1 | 2 | 3,13 | 2 |  |
| Q61753 | D-3-phosphoglycerate dehydrogenase | Phgdh | 2 | 14 | 3,12 | 2 | IvRsaT57 VgRagT78 LlReaS383 |
| P09055 | Integrin beta-1 | Itgb1 | 1 | 5 | 3,11 | 2 |  |
| P53996 | Cellular nucleic acid-binding protein | Cnbp | 0 | 6 | 3,10 | 2 | LaRecT173 |
| Q9CQE8 | UPF0568 protein C14orf166 homolog | RTRAF | 0 | 6 | 3,09 | 2 |  |
| Q9Z2I8 | Succinyl-CoA ligase [GDP-forming] subunit beta, mitochondrial | Suclg2 | 2 | 15 | 3,09 | 2 |  |
| Q9Z2I0 | LETM1 and EF-hand domain-containing protein 1, mitochondrial | Letm1 | 0 | 5 | 3,05 | 2 | LsRccT55 |
| Q91VW5 | Golgin subfamily A member 4 | Golga4 | 9 | 48 | 3,02 | 2 | LvRtsS119 LeRqrS891 LqRrlS1761 |
| Q9JHI7 | Exosome complex component RRP45 | Exosc9 | 1 | 6 | 3,02 | 2 |  |
| P80316 | T-complex protein 1 subunit epsilon | Cct5 | 4 | 12 | 3,01 | 2 |  |
| Q91YH5 | Atlastin-3 | Atl3 | 1 | 6 | 3,01 | 2 | LiRdwS216 |
| Q8K154 | UDP-glucuronosyltransferase | Ugt2b34 | 1 | 7 | 3,01 | 2 | LgRptT248 |
| Q8R081 | Heterogeneous nuclear ribonucleoprotein L | Hnrnpl | 0 | 4 | 3,00 | 2 | VdRaiT434 VkRptS528 |
| A0A087WQ89 | MCG5930 | 2210011C24Rik | 1 | 9 | 2,97 | 2 | LsRpgS48 |
| Q61029 | Lamina-associated polypeptide 2, isoforms beta/delta/epsilon/gamma | Tmpo | 14 | 24 | 2,96 | 2 | VgRkaT95 LtResT250 |
| Q8CGC7 | Bifunctional glutamate/proline--tRNA ligase;Glutamate--tRNA ligase;Proline--tRNA ligase | Eprs | 0 | 9 | 2,96 | 2 | LaRiaT63 LrRgmT467 IlRpwS1053 |
| Q3UEP4 | UDP-glucuronosyltransferase | Ugt2b36 | 1 | 6 | 2,96 | 2 | VlRpsT60 LgRptT246 |
| P32067 | Lupus La protein homolog | Ssb | 4 | 11 | 2,95 | 2 | LnRltT63 IrRspS94 |
| Q0VG62 | Uncharacterized protein C8orf59 homolog | 1810022K09Rik | 1 | 2 | 2,95 | 2 | VpRpeT34 |
| E9Q5F4 | Actin, cytoplasmic 1 | Actb | 30 | 31 | 2,94 | 2 |  |
| Q91ZA3 | Propionyl-CoA carboxylase alpha chain, mitochondrial | Pcca | 0 | 9 | 2,94 | 2 | VsRslS48 ViRgvT475 |
| P1063 | Thioredoxin | Txn | 1 | 3 | 2,92 | 2 |  |
| Q6VGS5 | Protein Daple | Ccdc88c | 0 | 19 | 2,91 | 2 | LqRelS787 LiRqhS1184 VdRtdT1493 VsRsaS1792 LsRafS1798 LaRerT1848 |
| P24369 | Peptidyl-prolyl cis-trans isomerase B | Ppib | 0 | 5 | 2,90 | 2 |  |
| Q6ZWQ0 | Nesprin-2 | Syne2 | 12 | 67 | 2,90 | 2 | IrRgrT968 LrRlmS1184 LaRlqT3013 IiRklS3097 LqRvrS3527 LsRtnS4096 VkRlyS4634 LhRlqT4927 IsRlvT5687 LqRwrT5743 IsRlqS5794 LaRieS6030 LlRqgT6489 |
| Q8CGB3 | Uveal autoantigen with coiled-coil domains and ankyrin repeats | Uaca | 0 | 13 | 2,90 | 2 |  |
| Q9QUI0 | Transforming protein RhoA | Rhoa | 0 | 2 | 2,89 | 2 |  |
| P56391 | Cytochrome c oxidase subunit 6B1 | Cox6b1 | 1 | 2 | 2,88 | 2 |  |
| P14602 | Heat shock protein beta-1 | Hspb1 | 2 | 5 | 2,87 | 2 | LlRspS15 LnRqlS86 IsRcfT143 |
| Q791V5 | Mitochondrial carrier homolog 2 | Mtch2 | 1 | 5 | 2,86 | 2 |  |
| Q9DBF1 | Alpha-aminoadipic semialdehyde dehydrogenase | Aldh7a1 | 3 | 9 | 2,85 | 2 | LgRlvS133 VdRlrS352 |
| Q3U0V1 | Far upstream element-binding protein 2 | Khsrp | 2 | 7 | 2,84 | 2 |  |
| P97432 | Next to BRCA1 gene 1 protein | Nbr1 | 0 | 2 | 2,82 | 2 | LgRpeS351 ViRslT659 |
| P52480 | Pyruvate kinase PKM | Pkm | 1 | 2 | 2,82 | 2 | LvRasS403 |
| A2AI08 | Taperin | Tprn | 2 | 8 | 2,82 | 2 | VnRslS255 |
| P68373 | Tubulin alpha-1C chain | Tuba1c | 7 | 18 | 2,80 | 2 | LnRliS232 |
| P10649 | Glutathione S-transferase Mu 1 | Gstm1 | 1 | 7 | 2,78 | 2 |  |
| Q9WU79 | Proline dehydrogenase 1, mitochondrial | Prodh | 1 | 6 | 2,78 | 2 |  |
| Q99PP6 | Tripartite motif-containing protein 34A | Trim34a | 2 | 8 | 2,77 | 2 | LrRewS243 |
| Q8BX02 | KN motif and ankyrin repeat domain-containing protein 2 | Kank2 | 0 | 4 | 2,76 | 2 |  |
| P53395 | Lipoamide acyltransferase  component of branched-chain alpha-keto acid dehydrogenase complex, mitochondrial | Dbt | 1 | 10 | 2,75 | 2 | VlRtwS11 |
| P62835 | Ras-related protein Rap-1A | Rap1a | 1 | 4 | 2,74 | 2 |  |
| Q8JZN5 | Acyl-CoA dehydrogenase family member 9, mitochondrial | Acad9 | 1 | 3 | 2,74 | 2 | LsRgaT12 VlRefT28 |
| Q8C052 | Microtubule-associated protein 1S | Map1s | 0 | 6 | 2,74 | 2 | LrRllS324 LaRrsT659 LtRkpS831 |
| P51658 | Estradiol 17-beta-dehydrogenase 2 | Hsd17b2 | 2 | 7 | 2,73 | 2 |  |
| P19536 | Cytochrome c oxidase subunit 5B, mitochondrial | Cox5b | 1 | 2 | 2,70 | 2 |  |
| Q9D6Y9 | 1,4-alpha-glucan-branching enzyme | Gbe1 | 1 | 7 | 2,69 | 2 |  |
| Q99MR8 | Methylcrotonoyl-CoA carboxylase subunit alpha, mitochondrial | Mccc1 | 1 | 10 | 2,69 | 2 |  |
| Q61037 | Tuberin | Tsc2 | 5 | 23 | 2,68 | 2 | IlRelS49 LlRadS625 IyRcaS802 LvRrpT1203 LpRsnT1270 LhRsvS1291 IeRaiS1365 |
| Q8QZY3 | Developmental pluripotency-associated protein 3 | Dppa3 | 0 | 8 | 2,68 | 2 |  |
| Q3TDQ1 | Dolichyl-diphosphooligosaccharide--protein glycosyltransferase subunit STT3B | Stt3b | 0 | 4 | 2,67 | 2 | ViRfeS94 |
| Q9R0Q7 | Prostaglandin E synthase 3 | Ptges3 | 0 | 2 | 2,64 | 2 |  |
| Q80U72 | Protein scribble homolog | Scrib | 1 | 10 | 2,63 | 2 | LqRraT475 LiRkdT606 IdRelS1218 LqRgpS1271 |
| ;Q8R3L2 | Transcription factor 25 | Tcf25 | 1 | 6 | 2,63 | 2 |  |
| Q91WQ3 | Tyrosine--tRNA ligase;Tyrosine--tRNA ligase, cytoplasmic;Tyrosine--tRNA ligase, cytoplasmic, N-terminally processed | Yars | 1 | 10 | 2,62 | 2 | VyRlsS138 |
| Q8K2I2 | Coiled-coil alpha-helical rod protein 1 | Cchcr1 | 0 | 5 | 2,62 | 2 | VeRmsT401 VaRipS459 |
| P35456 | Urokinase plasminogen activator surface receptor | Plaur | 1 | 4 | 2,60 | 2 |  |
| Q9QYC0 | Alpha-adducin | Add1 | 1 | 5 | 2,59 | 2 | VdRgsT212 |
| P28843 | Dipeptidyl peptidase 4 | Dpp4 | 1 | 5 | 2,59 | 2 |  |
| Q9R0L6 | Pericentriolar material 1 protein | Pcm1 | 5 | 30 | 2,59 | 2 | VgRrrT77 LtReiS378 VsRhiS1432 |
| Q922B9 | Sperm-specific antigen 2 homolog | Ssfa2 | 1 | 4 | 2,59 | 2 | LqRigS268 LtRsnT290 LlRtaS502 LpReeS640 VdRsqS728 LiReqS1075 |
| G3UWQ7 | Protein regulator of cytokinesis 1 | Prc1 | 0 | 2 | 2,59 | 2 | LqRelS601 |
| Q8C4X2 | Casein kinase I isoform gamma-3 | Csnk1g3 | 0 | 11 | 2,58 | 2 |  |
| Q9Z0P5 | Twinfilin-2 | Twf2 | 1 | 4 | 2,57 | 2 | LfRldS75 |
| P16331 | Phenylalanine-4-hydroxylase | Pah | 0 | 9 | 2,56 | 2 | LsRklS16 IpRpfS411 |
| Q9WVK4 | EH domain-containing protein 1 | Ehd1 | 1 | 6 | 2,56 | 2 |  |
| Q9Z1M8 | Protein Red | Ik | 0 | 5 | 2,54 | 2 |  |
| P46978 | Dolichyl-diphosphooligosaccharide--protein glycosyltransferase subunit STT3A | Stt3a | 1 | 5 | 2,54 | 2 | VlRfeS43 VdRegS631 |
| P21278 | Guanine nucleotide-binding protein subunit alpha-11 | Gna11 | 1 | 6 | 2,53 | 2 | VdRiaT169 |
| O70194 | Eukaryotic translation initiation factor 3 subunit D | Eif3d | 1 | 5 | 2,51 | 2 | VqRvgS274 ViRvyS521 |
| P47740 | Fatty aldehyde dehydrogenase | Aldh3a2 | 2 | 11 | 2,49 | 2 |  |
| O70479 | BTB/POZ domain-containing adapter for CUL3-mediated RhoA degradation protein 2 | Tnfaip1 | 1 | 7 | 2,49 | 2 | LtRhdT51 VkRysT302 |
| Q8BG05 | Heterogeneous nuclear ribonucleoprotein A3 | Hnrnpa3 | 0 | 5 | 2,46 | 2 | VsRedS94 |
| Q8BJ64 | Choline dehydrogenase, mitochondrial | Chdh | 2 | 3 | 2,44 | 2 | VsRgkT196 |
| P24456 | Cytochrome P450 2D10 | Cyp2d10 | 3 | 10 | 2,42 | 2 | LpRitS382 |
| Q9WV55 | Vesicle-associated membrane protein-associated protein A | Vapa | 1 | 7 | 2,41 | 2 |  |
| B7ZMP1 | Probable Xaa-Pro aminopeptidase 3 | Xpnpep3 | 1 | 3 | 2,41 | 2 | LqRryS32 |
| P28656 | Nucleosome assembly protein 1-like 1 | Nap1l1 | 1 | 6 | 2,40 | 2 |  |
| Q9QXG4 | Acetyl-coenzyme A synthetase, cytoplasmic | Acss2 | 0 | 3 | 2,40 | 2 |  |
| Q91VH2 | Sorting nexin-9 | Snx9 | 1 | 9 | 2,39 | 2 | IqRgnS194 VkRvgT543 |
| P18406 | Protein CYR61 | Cyr61 | 1 | 10 | 2,39 | 2 | IcRaqS95 |
| Q9DAW6 | U4/U6 small nuclear ribonucleoprotein Prp4 | Prpf4 | 2 | 6 | 2,38 | 2 |  |
| Q8BP47 | Asparagine--tRNA ligase, cytoplasmic | Nars | 2 | 6 | 2,38 | 2 | VlRdgT164 LeRflS536 |
| Q9R0H0 | Peroxisomal acyl-coenzyme A oxidase 1 | Acox1 | 3 | 13 | 2,37 | 2 |  |
| P24549 | Retinal dehydrogenase 1 | Aldh1a1 | 3 | 15 | 2,35 | 2 |  |
| Q64458 | Cytochrome P450 2C29 | Cyp2c29 | 2 | 9 | 2,33 | 2 | LwRqsS24 VgRhrS336 |
| P61967 | AP-1 complex subunit sigma-1A | Ap1s1 | 0 | 3 | 2,31 | 2 |  |
| P16406 | Glutamyl aminopeptidase | Enpep | 1 | 8 | 2,28 | 2 | ViRyiS849 LgRivT885 |
| Q922M3 | BTB/POZ domain-containing adapter for CUL3-mediated RhoA degradation protein 3 | Kctd10 | 2 | 12 | 2,27 | 2 |  |
| Q62433 | Protein NDRG1 | Ndrg1 | 7 | 10 | 2,11 | 2 | LmRsrT328 |
| P62960 | Nuclease-sensitive element-binding protein 1 | Ybx1 | 7 | 20 | 2,00 | 2 | InRndT78 |
| Q99JB8 | Protein kinase C and casein kinase II substrate protein 3 | Pacsin3 | 4 | 19 | 2,61 | 1 |  |
| Q9QXZ0 | Microtubule-actin cross-linking factor 1 | Macf1 | 15 | 56 | 2,47 | 1 | LeRekS418 IkRkyT814 VfRskT854 IeRnqT1331 IdRqvT1767 LkRqgS3889 IsRqkS4129 LeRrwT5394 LsRgdS6362 IeRgrS6501 |
| Q9CY66 | H/ACA ribonucleoprotein complex subunit 1 | Gar1 | 2 | 3 | 2,26 | 1 |  |
| P60766 | Cell division control protein 42 homolog | Cdc42 | 0 | 3 | 2,25 | 1 |  |
| Q9DB77 | Cytochrome b-c1 complex subunit 2, mitochondrial | Uqcrc2 | 4 | 10 | 2,18 | 1 | LsRagS9  LlRlaS87 |
| Q03963 | Interferon-induced, double-stranded RNA-activated protein kinase | Eif2ak2 | 0 | 4 | 2,11 | 1 |  |
| Q99N93 | 39S ribosomal protein L16, mitochondrial | Mrpl16 | 1 | 3 | 2,07 | 1 |  |
| QQ80YE7 | Death-associated protein kinase 1 | Dapk1 | 1 | 9 | 2,04 | 1 | IeRevS66 LsRkaS289 VsRrdS1433 |
| P68368 | Tubulin alpha-4A chain | Tuba4a | 7 | 18 | 2,01 | 1 | LnRliS232 |
| P42932 | T-complex protein 1 subunit theta | Cct8 | 5 | 18 | 1,97 | 1 | LvRlnS317 VlRgsT381 |
| Q9D0M3 | Cytochrome c1, heme protein, mitochondrial | Cyc1 | 3 | 4 | 1,96 | 1 |  |
| P50544 | Very long-chain specific acyl-CoA dehydrogenase, mitochondrial | Acadvl | 2 | 17 | 1,93 | 1 | LpRvaS208 |
| Q9DBG6 | Dolichyl-diphosphooligosaccharide--protein glycosyltransferase subunit 2 | Rpn2 | 0 | 5 | 1,91 | 1 | LdRpfT46 |
| Q8C7U1 | NEDD4-binding protein 3 | N4bp3 | 0 | 6 | 1,91 | 1 |  |
| O88967 | ATP-dependent zinc metalloprotease YME1L1 | Yme1l1 | 0 | 6 | 1,91 | 1 |  |
| E9Q555 | E3 ubiquitin-protein ligase RNF213 | Rnf213 | 8 | 24 | 1,90 | 1 | IhRggS427 LdRifS511 LfRtwT1602 LrRclT1845 IlRleS2501 VlRnfS2949 LyRkvS3210 LsRmgS3384 LlRdaS4057 LvRklS4233 LtRllT4537 LlRvqS4605 IqRqiS4908 |
| Q8CD15 | Bifunctional lysine-specific demethylase and histidyl-hydroxylase MINA | Mina | 2 | 9 | 1,90 | 1 | VcRsiS107 VtRklS309 |
| Q99L45 | Eukaryotic translation initiation factor 2 subunit 2 | Eif2s2 | 4 | 16 | 1,89 | 1 | VvRvgT212 |
| Q3TGW2 | Endonuclease/exonuclease/phosphatase family domain-containing protein 1 | Eepd1 | 3 | 8 | 1,87 | 1 | IpRdpS16 VfRlaT265 |
| Q8R0X7 | Sphingosine-1-phosphate lyase 1 | Sgpl1 | 2 | 14 | 1,87 | 1 |  |
| P97449 | Aminopeptidase N | Anpep | 12 | 28 | 1,87 | 1 | ViRmlS487 VnRppT745 LnRylS845 VtRrfS909 |
| P19157 | Glutathione S-transferase P 1 | Gstp1 | 2 | 7 | 1,86 | 1 |  |
| Q6R0H7 | Guanine nucleotide-binding protein G(s) subunit alpha isoforms XLas | Gnas | 5 | 15 | 1,84 | 1 | LpRshT699 |
| Q640L3 | Cell cycle progression protein 1 | Ccpg1 | 2 | 3 | 1,82 | 1 | LeRcwT286 |
| P80318 | T-complex protein 1 subunit gamma | Cct3 | 6 | 25 | 1,81 | 1 | IsRwsS170 LlRgaS380 |
| P49813 | Tropomodulin-1 | Tmod1 | 2 | 11 | 1,80 | 1 |  |
| Q8JZQ9 | Eukaryotic translation initiation factor 3 subunit B | Eif3b | 2 | 5 | 1,77 | 1 | VeRrrT741 |
| O70589 | Peripheral plasma membrane protein CASK | Cask | 1 | 5 | 1,75 | 1 | LkReaS64 LkRilT433 IhRqgT539 |
| Q9CPR4 | 60S ribosomal protein L17 | Rpl17 | 3 | 15 | 1,75 | 1 |  |
| P09411 | Phosphoglycerate kinase 1 | Pgk1 | 3 | 10 | 1,74 | 1 |  |
| O09167 | 60S ribosomal protein L21 | Rpl21 | 6 | 7 | 1,71 | 1 |  |
| Q69ZX8 | Actin-binding LIM protein 3 | Ablim3 | 3 | 11 | 1,67 | 1 | LhRtpS598 LeRhlS649 |
| P10126 | Elongation factor 1-alpha 1 | Eef1a1 | 6 | 15 | 1,67 | 1 | VgRveT269 |
| Q80TP3 | E3 ubiquitin-protein ligase UBR5 | Ubr5 | 8 | 29 | 1,66 | 1 | LsRlgS293 LyRllT1254 LrRsgT1751 LaRayS1783 ViRqiS1790 LeRkrT1971 |
| P11983 | T-complex protein 1 subunit alpha | Tcp1 | 6 | 18 | 1,63 | 1 |  |
| Q0P678 | Zinc finger CCCH domain-containing protein 18 | Zc3h18 | 3 | 4 | 1,62 | 1 |  |
| Q9DB20 | ATP synthase subunit O, mitochondrial | Atp5o | 2 | 7 | 1,60 | 1 |  |
| Q3UQ44 | Ras GTPase-activating-like protein IQGAP2 | Iqgap2 | 3 | 14 | 1,59 | 1 | LdRkqS554 LkRknS1458 |
| P62082 | 40S ribosomal protein S7 | Rps7 | 4 | 14 | 1,53 | 1 |  |
| P63037 | DnaJ homolog subfamily A member 1 | Dnaja1 | 3 | 11 | 1,52 | 1 |  |
| Q64339 | Ubiquitin-like protein ISG15 | Isg15 | 4 | 6 | 1,50 | 1 |  |
| Q91YD6 | Villin-like protein | Vill | 7 | 14 | 1,49 | 1 |  |
| Q99K51 | Plastin-3 | Pls3 | 8 | 18 | 1,48 | 1 | LkRaeS339 LmRryT506 VnRtlS533 |
| P17225 | Polypyrimidine tract-binding protein 1 | Ptbp1 | 5 | 14 | 1,48 | 1 |  |
| P06151 | L-lactate dehydrogenase A chain | Ldha | 4 | 10 | 1,48 | 1 |  |
| Q62191 | E3 ubiquitin-protein ligase TRIM21 | Trim21 | 6 | 17 | 1,46 | 1 | LeRsgS260 |
| O08749 | Dihydrolipoyl dehydrogenase, mitochondrial | Dld | 2 | 9 | 1,46 | 1 |  |
| P26041 | Moesin | Msn | 8 | 21 | 1,45 | 1 |  |
| Q8VI94 | 2-5-oligoadenylate synthase-like protein 1 | Oasl1 | 8 | 20 | 1,45 | 1 | VlRstT80 |
| Q4U2R1 | E3 ubiquitin-protein ligase HERC2 | Herc2 | 22 | 54 | 1,42 | 1 | VyRakS97 LaRvgS198 LqRfqS347 LdRlaT406 LgRggS643 LlRqvS811 VaRriS1036 LlResT2123 LkRchS2773 LiRkkT2949 IpRqiT3041 LgRggS3191 VnRkpT3302 VnRivS3668 LrRllT3785 LgRggS4184 VkRsrS4432 |
| P62858 | 40S ribosomal protein S28 | Rps28 | 2 | 3 | 1,40 | 1 | LgRtgS23 |
| Q99KP6 | Pre-mRNA-processing factor 19 | Prpf19 | 2 | 11 | 1,36 | 1 |  |
| P46735 | Unconventional myosin-Ib | Myo1b | 78 | 83 | 1,34 | 1 | LeRdfS230 LeRafS325 LyRdlS539 LkRppT569 |
| … |  |  |  |  |  |  |  |
| Q9EP53 | Hamartin | Tsc1 | 6 | 16 | 0,71 | 0 | LfRnkS1094 |
