# Supplementary figures and images for: A phosphoproteomic approach reveals that PKD3 controls PKA-mediated glucose and tyrosine metabolism
Source: Life Sci Alliance. 2021 Jun 18;4(8):e202000863. doi: 10.26508/lsa.202000863 (PMC8321662; doi:10.26508/lsa.202000863)

FIGURE 4A PAH GAPDH  
Higher exposure

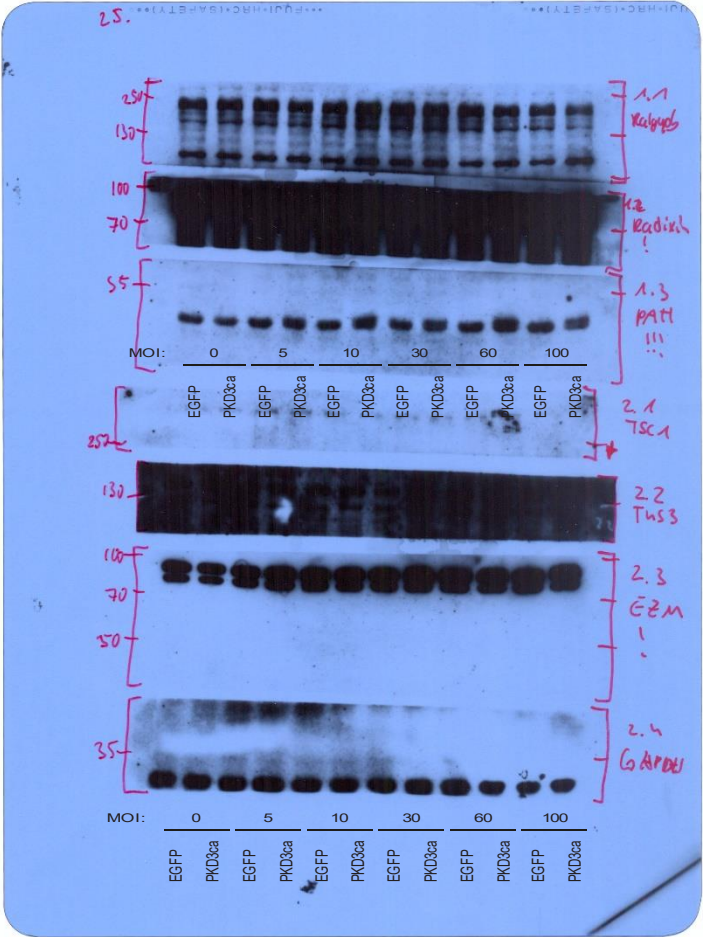

Lower exposure

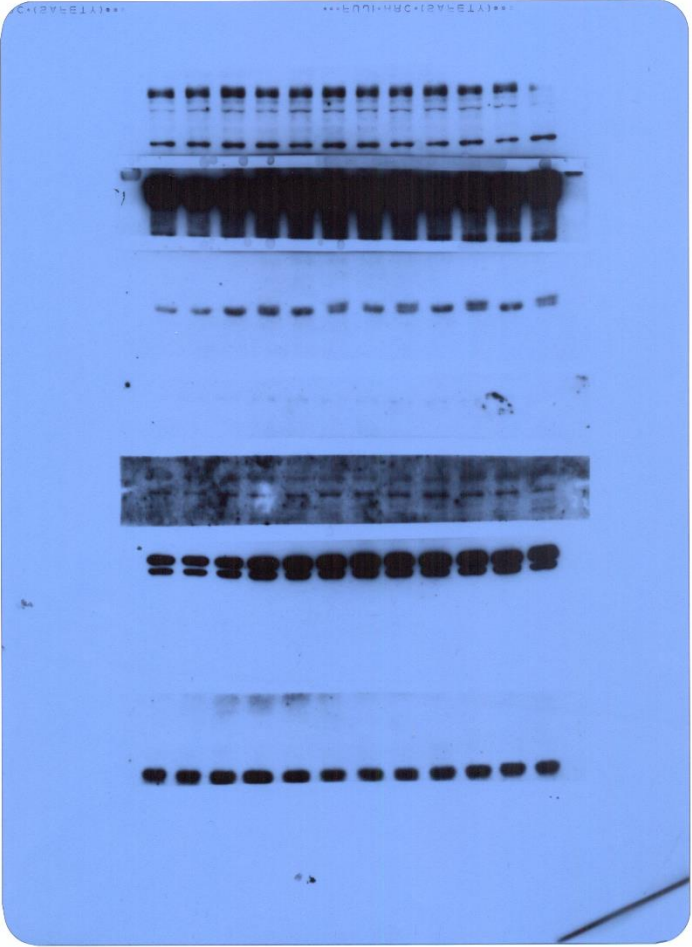

Figure 4F  
higher exposure

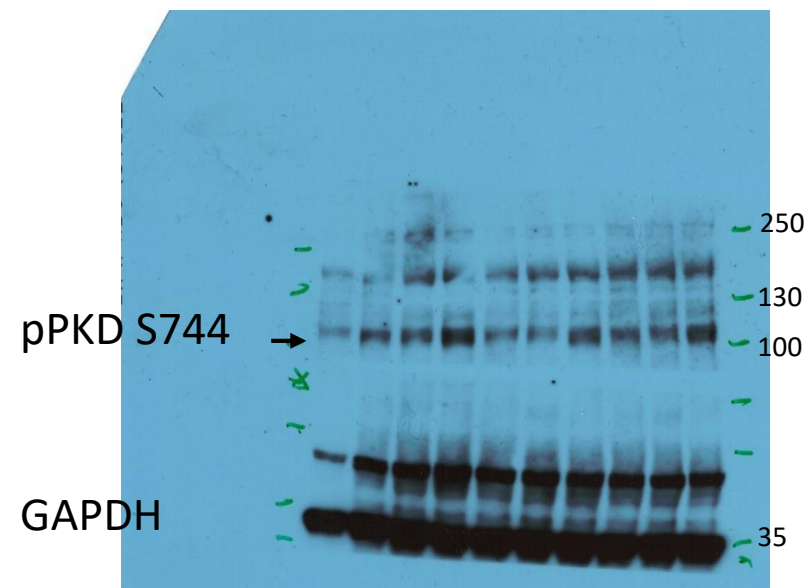

Lower exposure

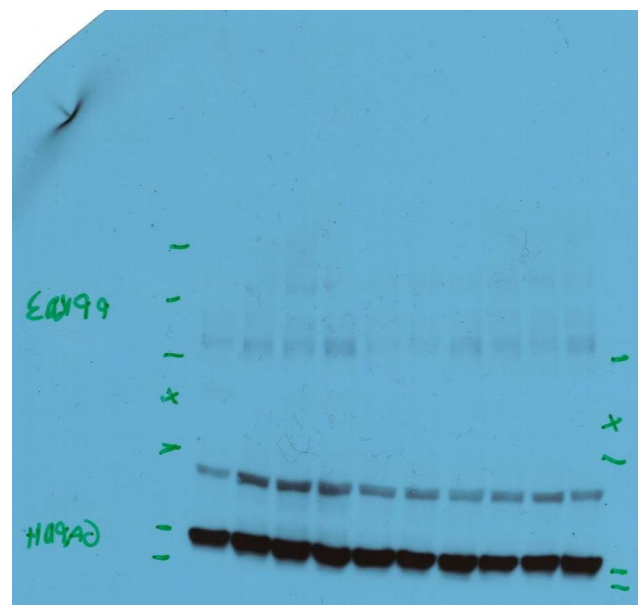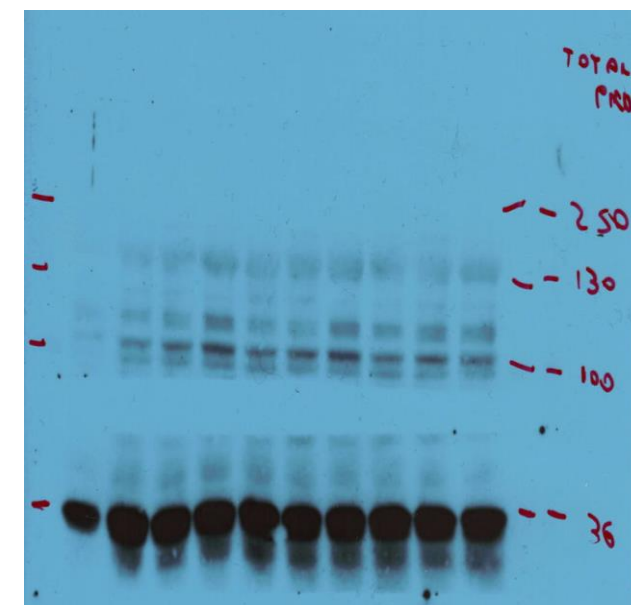

Supplement: Supplementary file 4 [file LSA-2020-00863_SdataF4.pdf]
