# Supplementary material for: A phosphoproteomic approach reveals that PKD3 controls PKA-mediated glucose and tyrosine metabolism
Source: Life Sci Alliance. 2021 Jun 18;4(8):e202000863. doi: 10.26508/lsa.202000863 (PMC8321662; doi:10.26508/lsa.202000863)
Supplement: Supplementary file 5 [file LSA-2020-00863_SdataF4_F5.pdf]

Figure 4A in vitro kinase assay

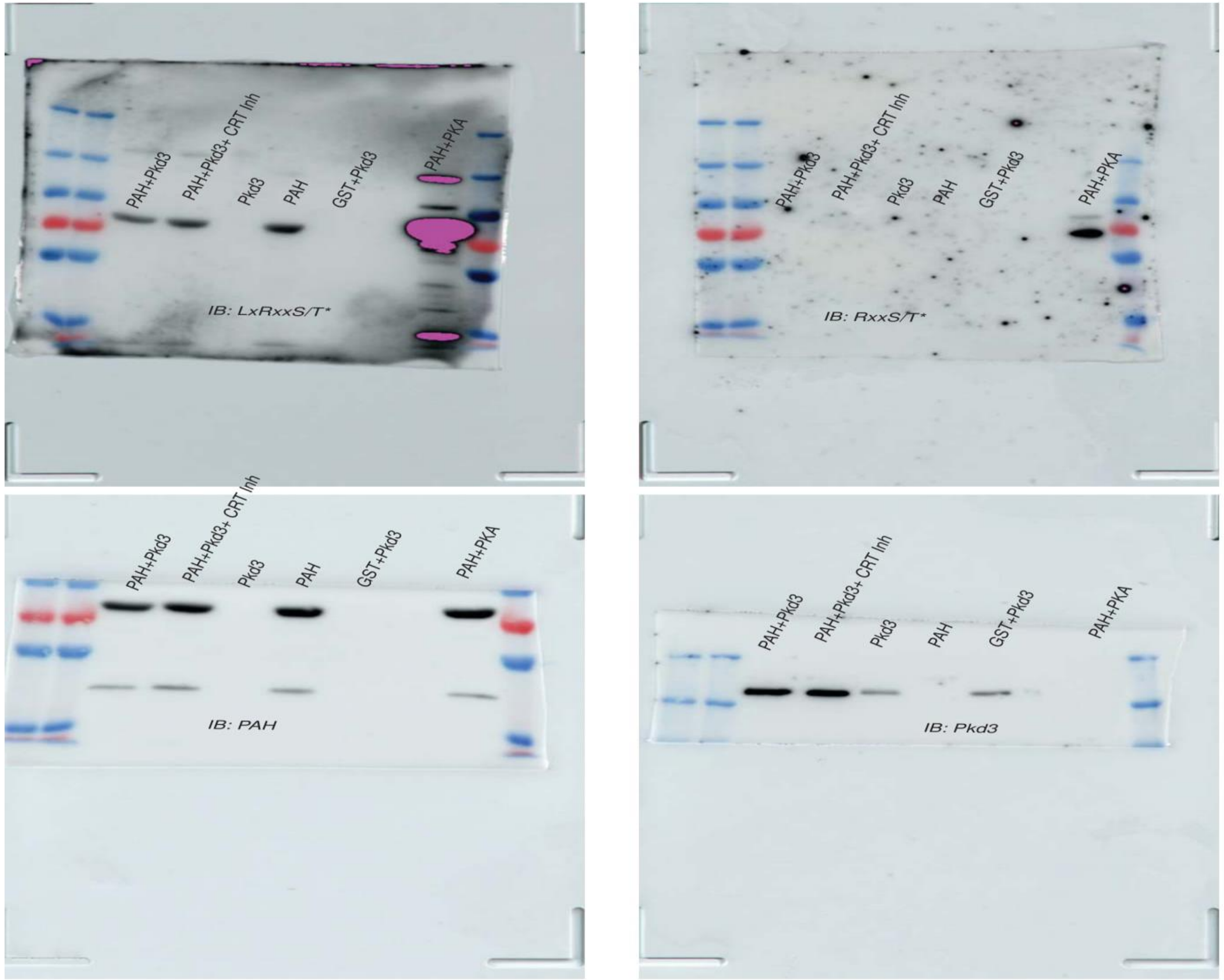

Figure 4A Overexpression of PKD3ca in primary hepatocytes

PKA substrate

Vinculin

pCREB

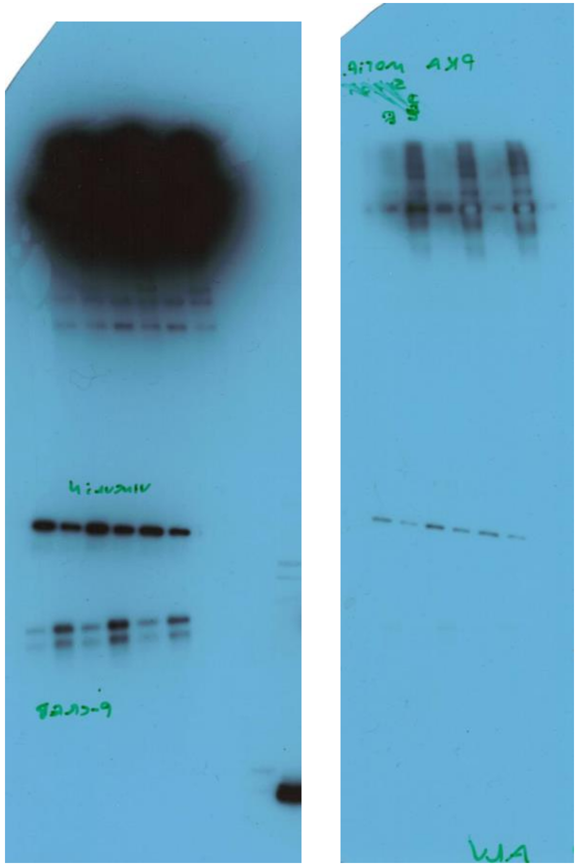

Figure 5C mice experiment DMSO CRT injected with glucagon 10 minutes Vinculin and pPKA thr 197

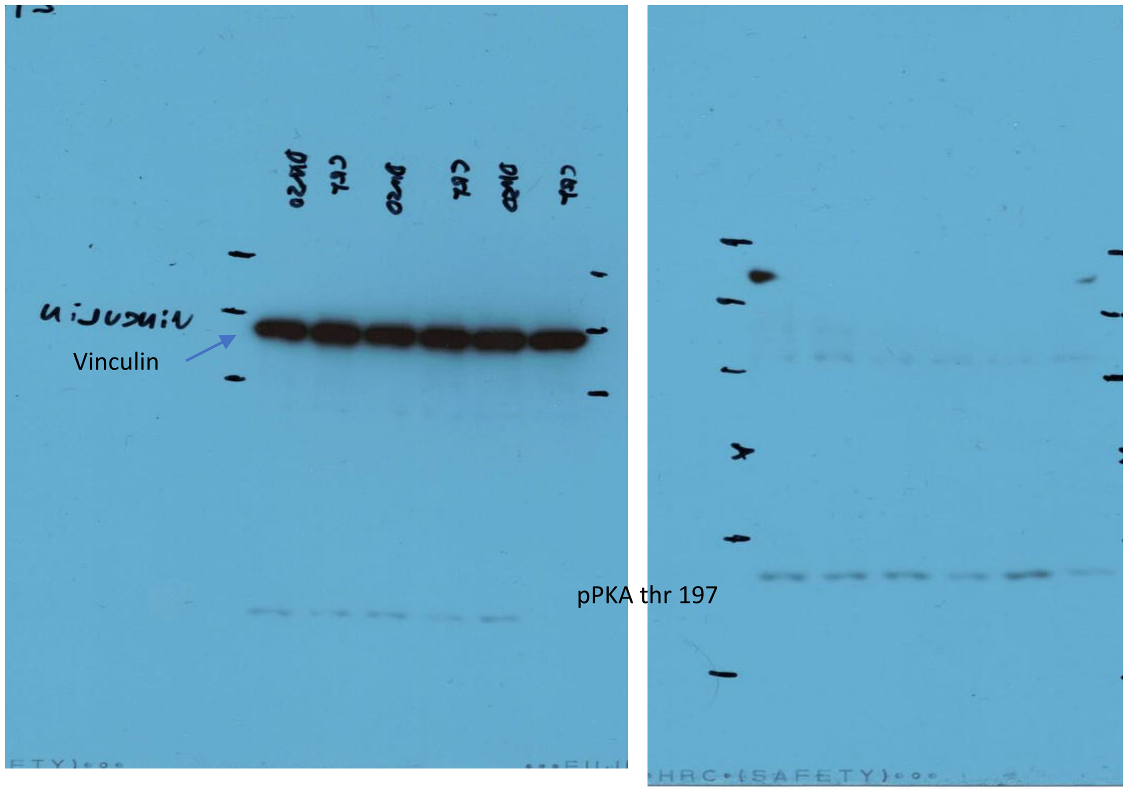

Figure 5C pPKD S744/748 and GAPDH

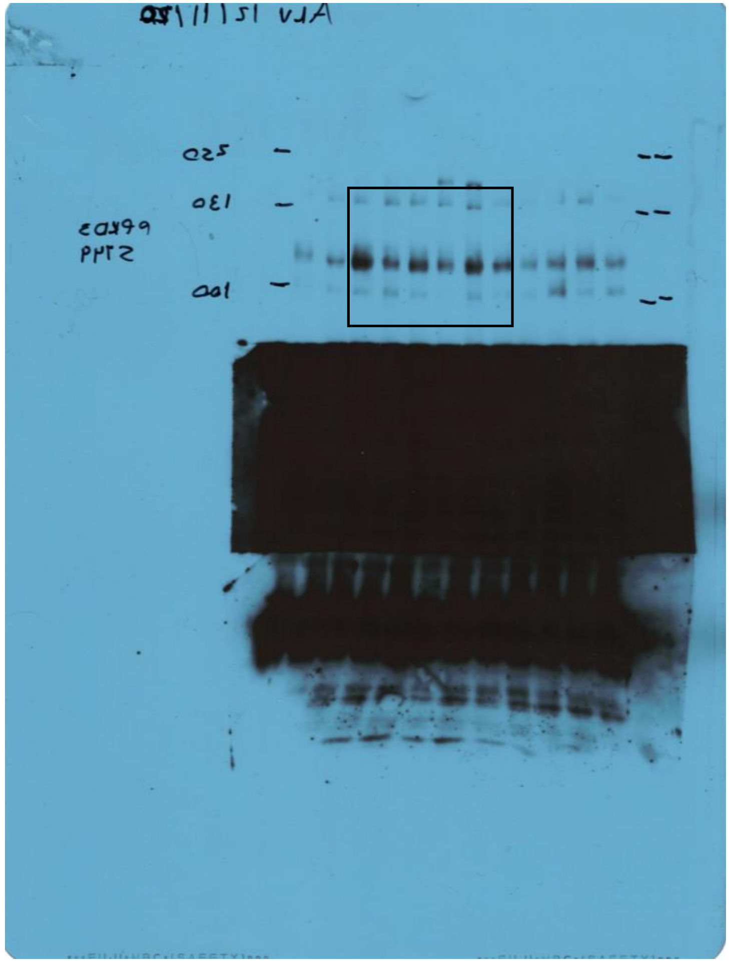

pPKD s744

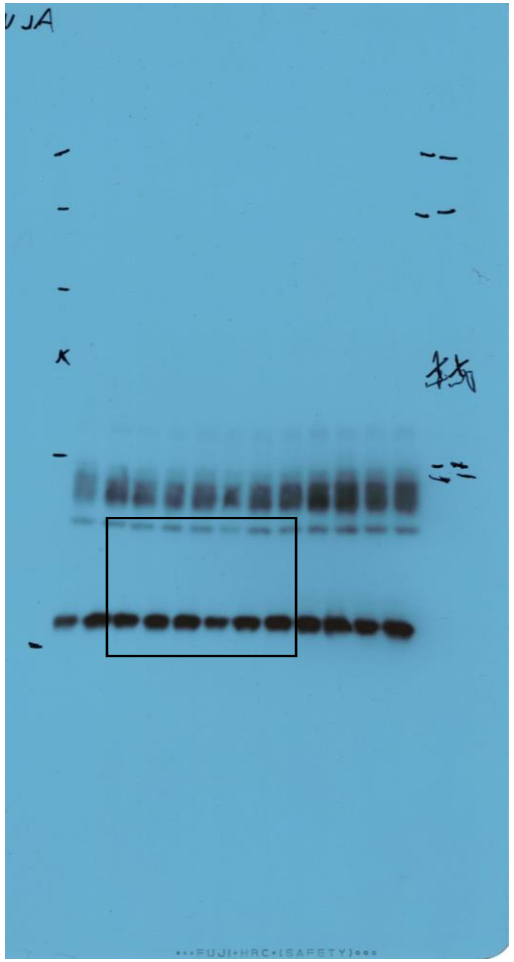

GAPDH

Figure 5D PKA substrate in mice treated with DMSO and CRT inhibitor

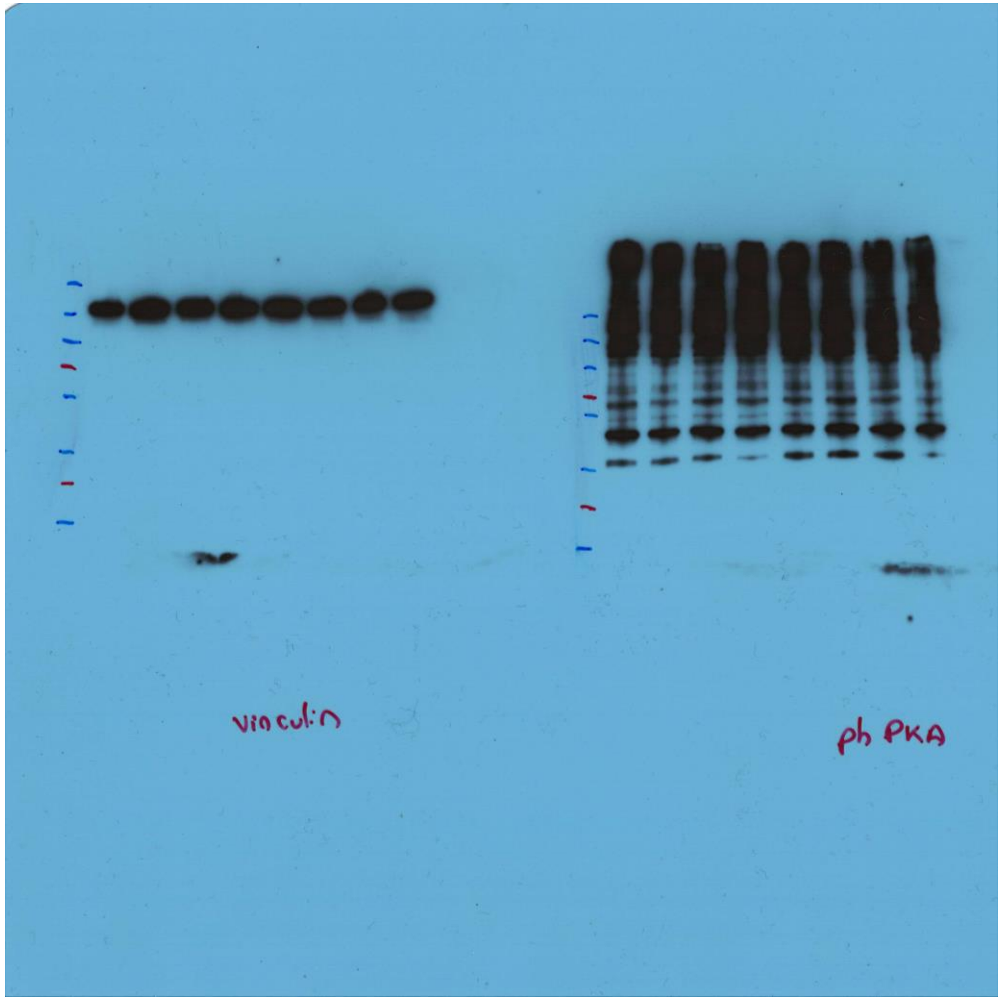

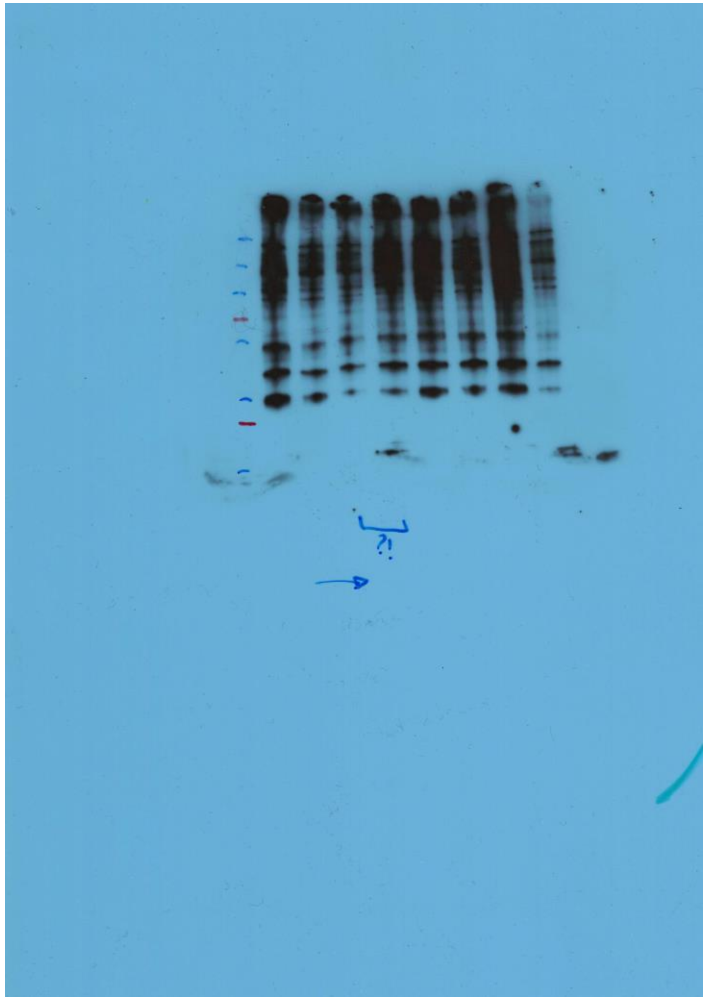

Figure 5 F-G

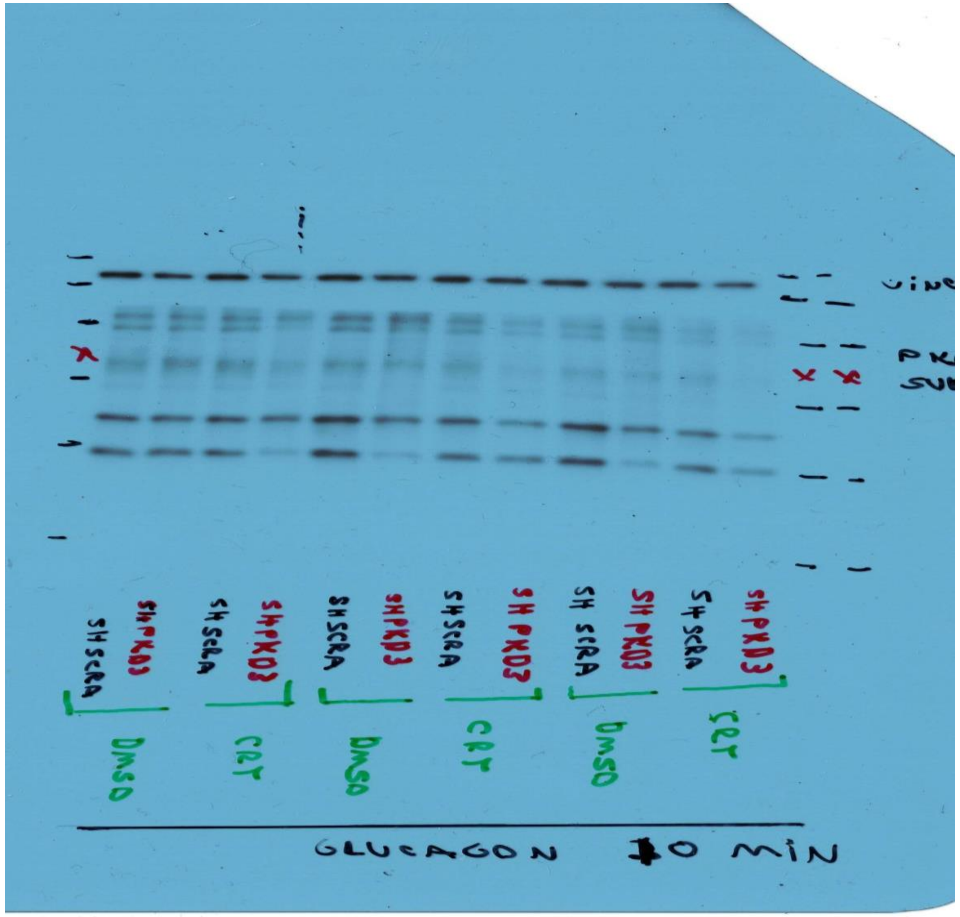

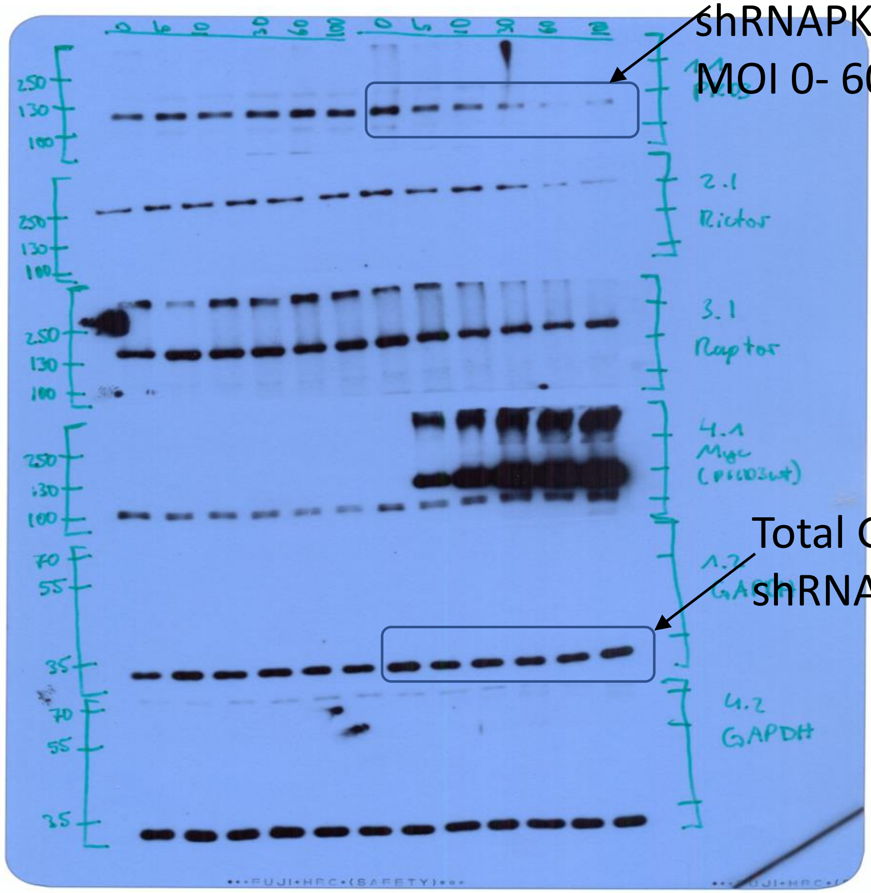

Total PKD3
shRNAPKD3 titration
MOI 0- 60

Total GAPDH
shRNAPKD3 titration
